# Rg1 protects H9C2 cells from high glucose‐/palmitate‐induced injury via activation of AKT/GSK‐3β/Nrf2 pathway

**DOI:** 10.1111/jcmm.15486

**Published:** 2020-06-16

**Authors:** Haitao Yu, Juan Zhen, Yang Yang, Jian Du, Jiyan Leng, Qian Tong

**Affiliations:** ^1^ The First Hospital of Jilin University Changchun China

**Keywords:** AKT/GSK‐3β/Nrf2, diabetes, ginsenoside Rg1, H9C2 cells

## Abstract

Our previous studies have assessed ginsenoside Rg1 (Rg1)‐mediated protection in a type 1 diabetes rat model. To uncover the mechanism through which Rg1 protects against cardiac injury induced by diabetes, we mimicked diabetic conditions by culturing H9C2 cells in high glucose/palmitate. Rg1 had no toxic effect, and it alleviated the high glucose/palmitate damage in a dose‐dependent manner, as indicated by 3‐(4,5‐dimethylthiazol‐2‐yl)‐2,5‐diphenyl tetrazolium bromide assay and lactate dehydrogenase release to the culture medium. Rg1 prevented high glucose/palmitate‐induced cell apoptosis, assessed using cleaved caspase‐3 and terminal deoxynucleotidyl transferase dUTP nick end labelling staining. Rg1 also reduced high glucose‐/palmitate‐induced reactive oxygen species formation and increased intracellular antioxidant enzyme activity. We found that Rg1 activates protein kinase B (AKT)/glycogen synthase kinase‐3 (GSK‐3β) pathway and antioxidant nuclear factor erythroid 2‐related factor 2 (Nrf2) pathway, indicated by increased phosphorylation of AKT and GSK‐3β, and nuclear translocation of Nrf2. We used phosphatidylinositol‐3‐kinase inhibitor Ly294002 to block the activation of the AKT/GSK‐3β pathway and found that it partially reversed the protection by Rg1 and decreased Nrf2 pathway activation. The results suggest that Rg1 exerts a protective effect against high glucose and palmitate damage that is partially AKT/GSK‐3β/Nrf2‐mediated. Further studies are required to validate these findings using primary cardiomyocytes and animal models of diabetes.

## 1 INTRODUCTION

Diabetic cardiomyopathy (DCM) is very common in patients with diabetes, the incidence of which is as high as 75%.^1^ However, as the occurrence and development of the disease are relatively insidious, the early symptoms are usually not obvious and easy to ignore. Early intervention of DCM is crucial to slow down the progress of disease and reduce mortality. The pathogenesis of DCM is very complex.^2^ Apoptosis caused by hyperglycemia is an important step in the pathogenesis of DCM.^3^ At present, it is known that the causes of cardiomyocyte apoptosis in DCM include hyperglycemia, hyperlipidemia, hypertension, oxidative stress and activation of the renin‐angiotensin system,^3^ but the specific mechanism is not clear.

Ginsenoside Rg1 (Rg1), one of the critical, active components of ginseng extract, has a wide range of physiological activities and significant medicinal value. It was found that Rg1 has a protective effect on various tissues and organs of the human body and is anti‐apoptotic, anti‐inflammatory and anti‐ageing.^4^, ^5^, ^6^ In our previous study, we found that Rg1 reduces the level of oxidative stress and apoptosis of cardiomyocytes in the myocardium of diabetic rats.^7^ However, the specific mechanism of prevention of DCM by Rg1 and the signal pathway involved are not clear.

Phosphatidylinositol‐3‐kinase (PI3K)/protein kinase B (AKT) signalling pathway is involved in cell proliferation, differentiation, apoptosis and glucose transport, which are closely related to the occurrence and development of DCM.^8^ Furthermore, several recent studies have shown that activation of the PI3K/AKT pathway may result in the up‐regulation of nuclear factor erythroid 2‐related factor 2 (Nrf2), which is an important antioxidant pathway.^9^, ^10^ However, it is not clear whether the protective effect of Rg1 is mediated by PI3K/AKT/Nrf2 signalling pathway. Therefore, we aimed to study the role of the PI3K/AKT signalling pathway in the prevention of high glucose and palmitate (G&P) damage by Rg1, and its relationship with PI3K/AKT signalling pathway and the activation of Nrf2.

In our study, we aimed to demonstrate that Rg1 could alleviate the G&P damage in a dose‐dependent manner and could protect against apoptosis and reactive oxygen species (ROS) production induced by G&P. We identified that Rg1 activates the AKT/GSK‐3β and Nrf2 pathways, which in turn protects H9C2 cell apoptosis, induced by G&P. Inhibition of the PI3K/AKT pathway by Ly294002 partially abolished the protection of Rg1 against G&P injury and down‐regulated Nrf2 expression. Thus, Rg1 provides a protective effect against G&P damage in H9C2 cells that is partially AKT/GSK‐3β/Nrf2‐mediated.

## 2 MATERIALS AND METHODS

### 2.1 Materials

Rg1 (purity > 98%) with high‐performance liquid chromatographic analysis was obtained from the Jilin University School of Pharmaceutical Sciences. Ly294002 was obtained from Cell Signaling Technology. The Cell Proliferation Kit (MTT) was obtained from Sigma.

### 2.2 Cell culture and treatment

H9C2 cells were maintained in Dulbecco's modified Eagle's medium (DMEM) supplemented with 10% foetal bovine serum (FBS), 2 mmol/L L‐glutamine and 100 U/mL penicillin at 37°C in a humidified chamber containing 5% CO_2_. When cell populations reached 40%‐50% confluence, the cultures were exposed to D‐glucose at a final concentration of 22.5 mmol/L (high glucose) and palmitate at a final concentration of 50 μmol/L for 24 hours. The dose of glucose and palmitate was based on a previous publication.^11^ In addition, some cultured cells were exposed to 5.5 mmol/L D‐glucose as control. After treatment, the monolayer cultures were collected with a gum rubber scraping device and lysed using lysis buffer. Rg1 pre‐treatment was performed by exposing cells to different doses of Rg1 (0, 5, 10, 20 and 40 μmol/L) for 2 hours and then incubating with G&P for another 24 hours. In one inhibition group, H9C2 cells were pre‐treated with 10 µmol/L Ly294002, a specific PI3K inhibitor (Cell Signaling Technology) at 37°C for 2 hours prior to the addition of Rg1, whereas the other inhibition group was treated with Ly294002 only.

### 2.3 Cell viability

H9C2 cells were seeded at a density of 5 × 10^3^ cells/well in 96‐well plates, and cell viability was determined using 3‐(4,5‐dimethylthiazol‐2‐yl)‐2,5‐diphenyl tetrazolium bromide (MTT) assay. The cells were incubated with G&P for 24 hours with or without pre‐treatment with various doses of Rg1 (0, 5, 10, 20 and 40 μmol/L) for 2 hours. Each well was washed twice with phosphate‐buffered saline (PBS) to remove the medium before 10% MTT was added to each well and incubated for an additional 4 hours at 37°C. The absorbance was measured using a microplate reader at 490 nm and used as a measurement of cell viability. The absorbance was normalized to cells incubated in the control medium, which were considered 100% viable.

### 2.4 Terminal deoxynucleotidyl transferase dUTP nick end labelling

We used the DeadEnd Colorimetric Terminal deoxynucleotidyl transferase dUTP nick end labelling (TUNEL) System (Promega) to determine the proportion of apoptotic cells according to the manufacturer's instructions. Briefly, H9C2 cells in different groups were fixed with 4% paraformaldehyde for 1 hour at room temperature and washed with PBS three times for 5 minutes each, and labelled with TdT (terminal deoxynucleotidyl transferase) reaction mix. After mounting with SlowFade Gold Antifade Mountant with DAPI (4′,6‐diamidino‐2‐phenylindole; Thermo Fisher Scientific), apoptotic cells (green) were detected by fluorescence microscopy. TUNEL‐positive cells were counted in ten fields per group, and the TUNEL‐positive cells/DAPI percentage was used as the ratio of apoptosis.

### 2.5 Lactate dehydrogenase release in culture medium

We used Pierce Lactate dehydrogenase (LDH) Cytotoxicity Assay Kit (Thermo Fisher Scientific) to determine the LDH release into the culture medium as previously described.^12^ Briefly, 50 µL of each sample medium was transferred to a 96‐well flat‐bottom plate in duplicate wells, mixed with 50 µL of the reaction mixture, then incubated at room temperature for 30 minutes in the dark, followed by the addition of 50 µL of Stop Solution to each sample well. Absorbance at 490 and 680 nm was measured using SpectraMax M Series Multi‐Mode Microplate Reader (Molecular Devices) to quantify signal (490 nm) and noise (680 nm) absorbance.

### 2.6 Detection of intracellular reactive oxygen species

Intracellular ROS levels were assessed using 2,7‐dichlorofluorescein diacetate (DCFHDA) according to the manufacturer's instructions (Nanjing Jiancheng Bioengineering Institute), which forms dichlorofluorescein, fluorescent compound with ROS. H9C2 cells were pre‐loaded with 10 μmol/L DCFH‐DA for 30 minutes at 37°C, and then the plates were washed with PBS three times. Fluorescence was determined using a microplate reader with excitation/emission wavelength at 485/525 nm.

### 2.7 Detection of malondialdehyde levels and Superoxide Dismutase, catalase and Glutathione peroxidase activity in H9C2 cells

H9C2 cells were cultured in 96‐well plates at 10^5^ cells/well. After various treatments, the cells were harvested to measure the malondialdehyde (MDA) levels, as well as the Superoxide Dismutase (SOD), catalase (CAT) and Glutathione peroxidase (GSH‐Px) activities in H9C2 cells with relevant commercial kits according to manufacturer's instructions (Nanjing Jiancheng Bioengineering Institute).

### 2.8 Histology

H9C2 cells were washed with cold PBS and then fixed with 4% paraformaldehyde for 20 minutes. Subsequently, cells were permeabilized with 0.2% Triton X‐100 and incubated with 5% bovine serum albumin to block non‐specific binding, and then incubated with anti‐Nrf2 (Abcam) overnight at 4°C in a humidified chamber. Then, the cells were incubated with Alexa Fluor 488‐labelled goat anti‐rabbit IgG antibody for 1 hour at 37°C, followed by incubation with DAPI (Thermo Fisher Scientific). Nuclear Nrf2 translocation was detected by fluorescence microscopy.

### 2.9 Western blotting

Proteins from total cell lysates were separated by sodium dodecyl sulphate‐polyacrylamide gel electrophoresis (SDS‐PAGE) and probed with different primary antibodies against phospho (p)‐AKT (Ser473; Cell Signaling Technology), AKT (Cell Signaling), p‐GSK‐3β (Ser256; Cell Signaling Technology), GSK‐3β (Cell Signaling Technology), Nrf2 (Abcam), HO‐1 (heme oxygenase‐1; Cell Signaling Technology), NQO‐1 (NAD(P)H quinone dehydrogenase 1; Cell Signaling) and GAPDH (glyceraldehyde‐3‐phosphate dehydrogenase; Abcam). Of note, we only showed cleaved‐caspase 3 bands in figures instead of total caspase 3 and cleaved‐caspase 3 bands in the same membrane; the reason for this is that when we exposure total‐caspase 3 and cleaved‐caspase 3 at the same time, the cleaved‐caspase 3 band was undetectable, which has been described in our previous study.^12^

### 2.10 Statistical analyses

Data are presented as mean ± standard error of the mean (SEM). Statistical differences were determined using two‐sided, unpaired Student's *t* tests or two‐way analysis of variance (ANOVA) followed by Tukey's multiple comparison test. *P* < .05 was considered statistically significant.

## 3 RESULTS

### 3.1 Protective effects of Rg1 against G&P‐induced H9C2 cell injury

**Figure 1:**
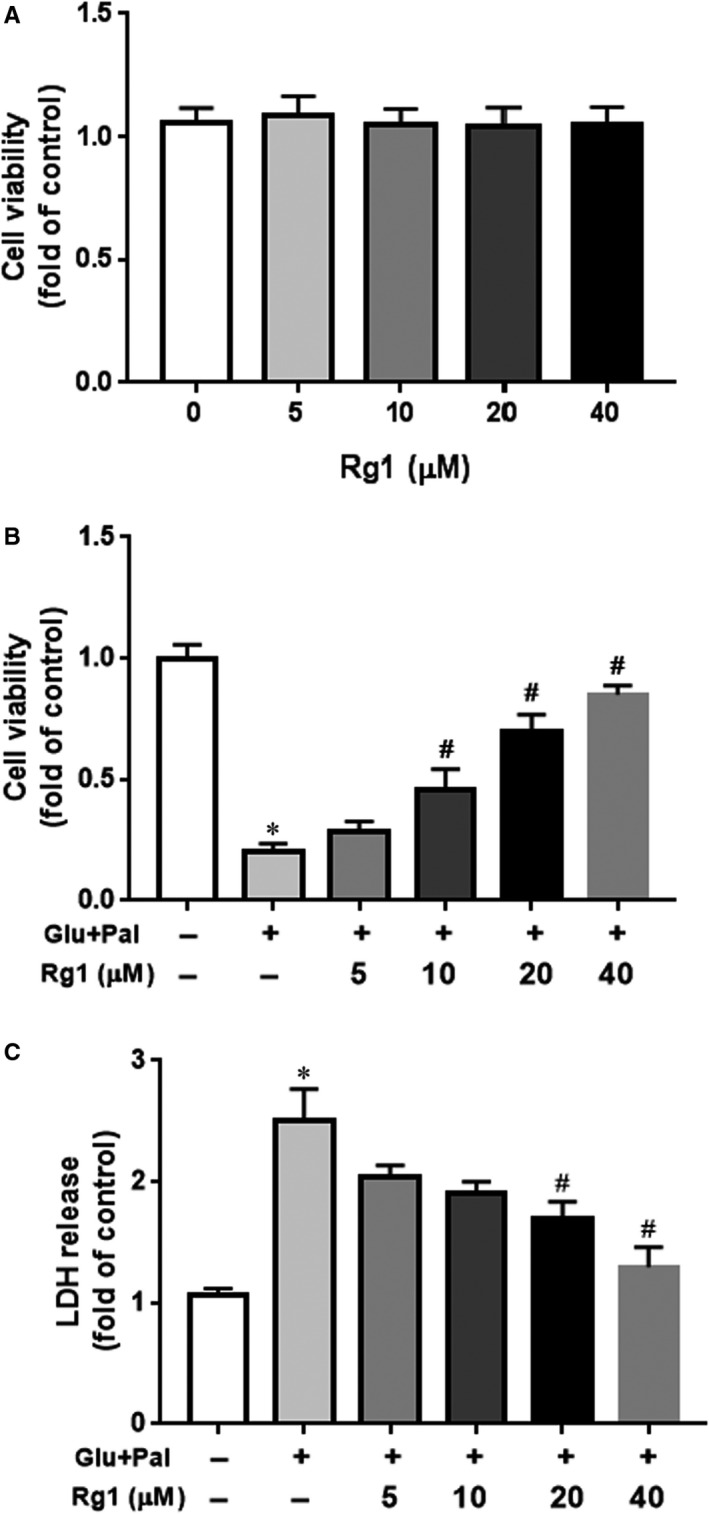
Protective effect of Rg1 on G&P‐induced‐H9C2 cell injury. Protective effects of ginsenoside Rg1 (Rg1) against high glucose and palmitate (G&P)‐induced H9C2 cell injury. A, H9C2 cells were treated with different doses of Rg1 (0, 5, 10, 20 and 40 µmol/L) for 24 h. Cell viability was tested using MTT (3‐(4,5‐dimethylthiazol‐2‐yl)‐2,5‐diphenyl tetrazolium bromide) assay; n = 5 per group. B, H9C2 cells were pre‐treated with different doses of Rg1 and co‐treated with G&P for another 24 h. Cell viability was tested by MTT assay; n = 5 per group. C, Lactate dehydrogenase (LDH) release in culture medium after pre‐treatment with different doses of Rg1 (0, 5, 10, 20 and 40 µmol/L) and then co‐treated with G&P for another 24 h; n = 5 per group. *P < .05 vs control group, ^#^ *P* < .05 vs Glucose (Glu) + Palmitate (Pal) group

We first assessed the toxicity of Rg1 on H9C2 cells, and we found no significant impact on cell viability after exposure to various doses of Rg1 (0, 5, 10, 20 and 40 µmol/L) for 24 hours (Figure 1A). We noted dose‐dependent protection of Rg1 against G&P injury indicated by cell viability and LDH release to the culture medium (Figure 1B,C).

### 3.2 Protective effects of Rg1 on G&P‐induced H9C2 cell apoptosis

**Figure 2:**
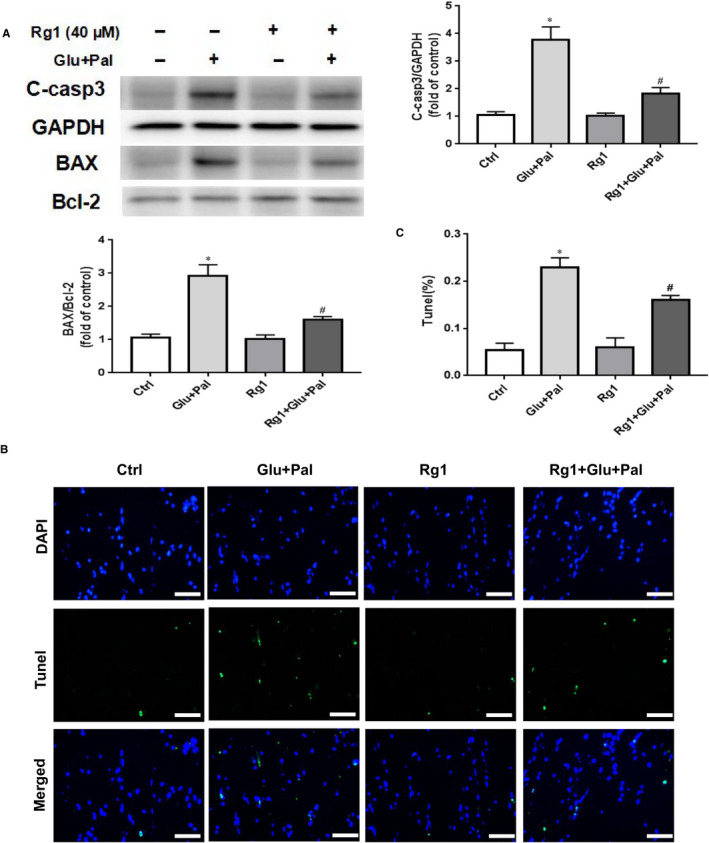
Protective effect of Rg1 on G&P‐induced H9C2 cell apoptosis. A, Cleaved caspase‐3, BAX and Bcl‐2 expression in each treatment group (control; Glu + Pal; Rg1; Rg1 + Glu +Pal); n = 5 per group. B, Representative immunofluorescent staining for terminal transferase dUTP nick end labelling assay (TUNEL, green) and nuclei (DAPI (4′,6‐diamidino‐2‐phenylindole), blue); 40× magnification. Scale bar = 50 μm. C, Summary of TUNEL data for each group (n = 5 per group). *P < .05 vs control group, #*P* < .05 vs Glu + Pal group

Many studies have demonstrated that apoptosis is the main cause of DCM. We evaluated the degree of involvement of apoptosis in G&P damage in H9C2 cells in vitro. We identified an increase in cleaved caspase‐3 and Bcl‐2‐associated X protein (BAX)/B‐cell lymphoma 2 (Bcl‐2) ratio after H9C2 cell treatment with G&P for 24 hours, as well as protective effects of Rg1 indicated by suppressed cleaved caspase‐3 expression and BAX/Bcl‐2 ratio (Figure 2A). In addition, G&P increased the TUNEL‐positive cell ratio, and Rg1 prevented G&P‐induced apoptosis (Figure 2B,C).

### 3.3 Rg1 reduced G&P‐induced ROS formation and increased intracellular antioxidant enzyme activity

**Figure 3:**
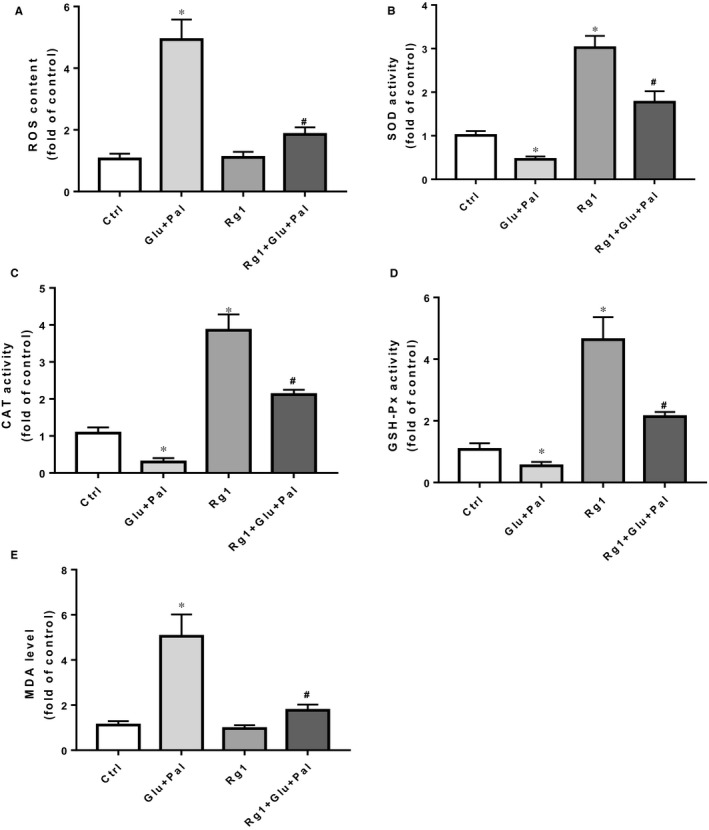
Rg1 reduced G&P‐induced ROS formation and increased intracellular antioxidant enzyme activity. Effects of Rg1 on G&P‐induced intracellular ROS formation (A), SOD activity (B), CAT activity (C), GSH‐Px activity (D) and MDA levels (E) of different groups in H9C2 cells. n = 5 per group. *P < .05 vs control group, ^#^ *P* < .05 vs Glu + Pal group

Oxidative stress plays a vital role in DCM, and extensive ROS is also a main cause of cell apoptosis.^1^ We examined the ROS and MDA levels in different groups, as well as intracellular antioxidant enzyme activity (SOD, CAT and GSH‐Px activities). We found that G&P significantly increased ROS levels in H9C2, and Rg1 inhibited G&P‐induced ROS production (Figure 3A). Compared with H9C2 cells in the control group, those in the non‐treated G&P control group had higher levels of MDA and lower levels of the antioxidants (SOD, CAT and GSH‐Px), and Rg1 significantly lowered the level of MDA induced by G&P (Figure 3E); moreover, Rg1 significantly increased the activity of SOD, CAT and GSH‐Px in H9C2 cells (Figure 3B‐D). This suggests that Rg1 is associated with reduced oxidative stress in H9C2 cells.

### 3.4 Effects of Rg1 on AKT/GSK‐3β and Nrf2 pathway in H9C2 cells

**Figure 4:**
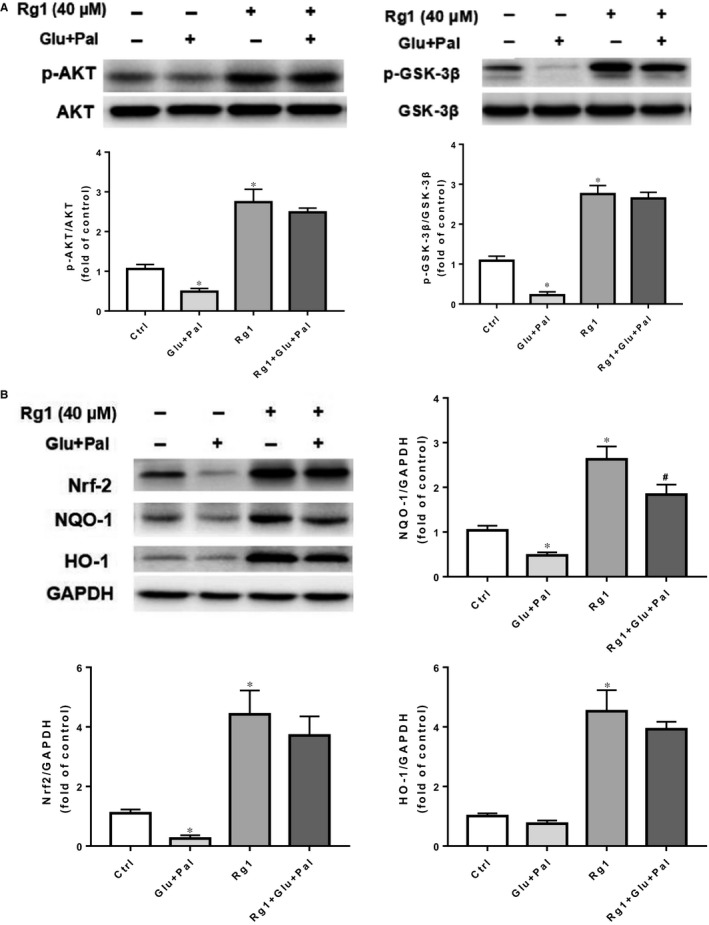
Effects of Rg1 on AKT/GSK‐3β and Nrf2 pathways in H9C2 cells. A, Expression of phospho (p)‐AKT, t‐AKT, p‐GSK‐3β, t‐GSK‐3β in different groups (control; Glu + Pal; Rg1; Rg1 + Glu +Pal) tested by Western blotting; n = 5 per group. B, Nrf2, HO‐1, NQO‐1 expression in each group (control; Glu + Pal; Rg1; Rg1 + Glu +Pal) tested by Western blotting; n = 5 per group. *P < .05 vs control group, #*P* < .05 vs Rg1 group

PI3K/AKT pathway plays an essential role in many cellular processes, including proliferation, apoptosis and cell migration.^8^ Previous studies have shown that this pathway is involved in high glucose‐induced apoptosis.^13^ The Nrf2 pathway has been shown to be up‐regulated during the antioxidative response to cellular stress, including high glucose exposure.^14^ Therefore, we evaluated whether the same is also involved in Rg1 protection on H9C2 cells exposed to G&P. We identified that G&P significantly decreased p‐AKT, p‐GSK‐3β, Nrf2, HO‐1, and NQO‐1 expression, and Rg1 (at 40 µmol/L) significantly induced p‐AKT, p‐GSK‐3β, Nrf2, HO‐1 and NQO‐1 expression (Figure 4A,B). We stained H9C2 cells of different groups to co‐localize Nrf2, and nuclear localization was observed by immunofluorescence to confirm if Nrf2 pathway was activated by Rg1. We found that Rg1 treatment increased Nrf2 nuclear translocation, and G&P had no influence on it (Figure S1).

### 3.5 Effects of a PI3K inhibitor on H9C2 cells exposed to Rg1 and/or G&P

**Figure 5:**
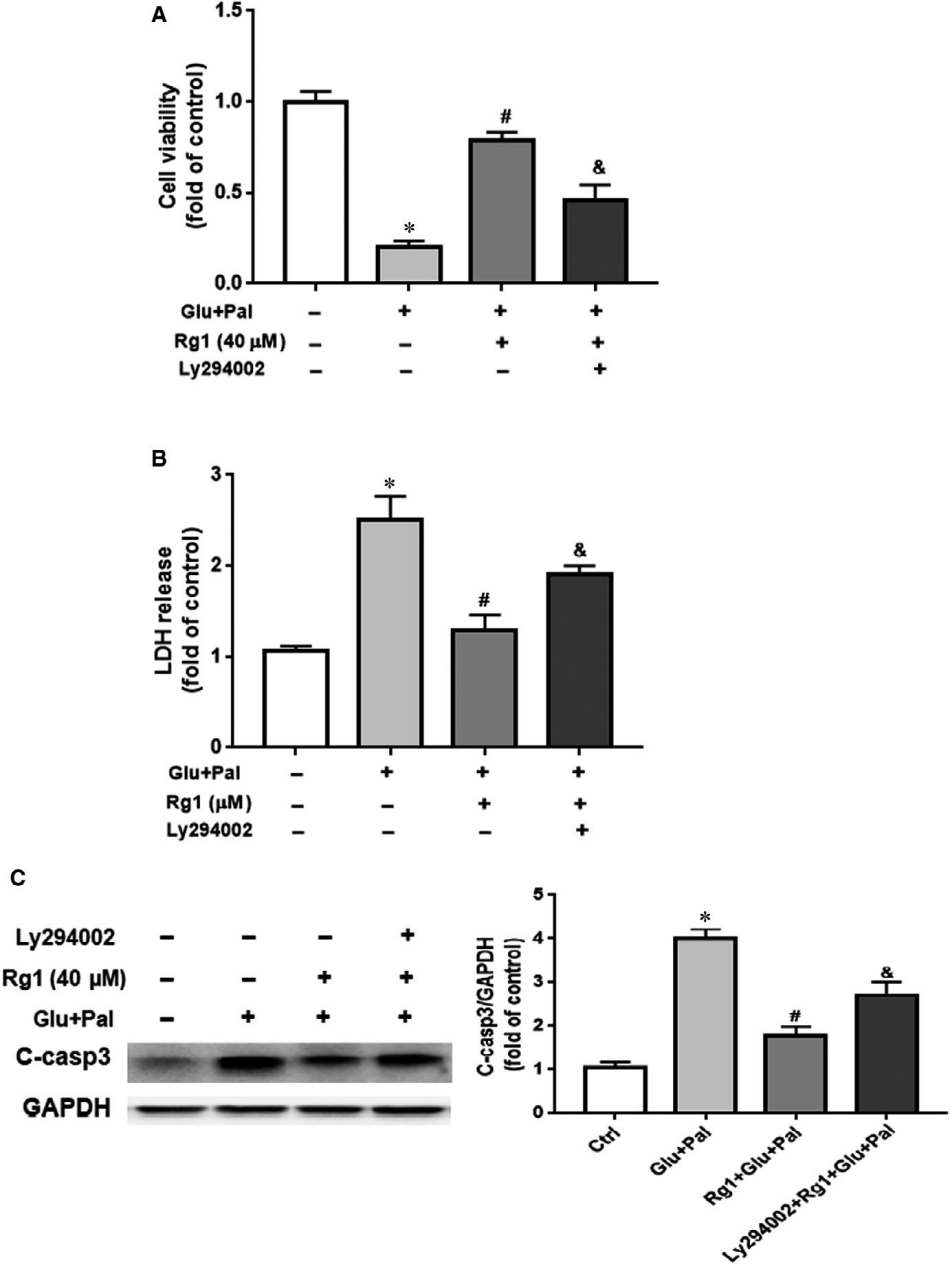
Effects of PI3K inhibitor Ly294002 on H9C2 cells exposed to Rg1 and/or G&P. H9C2 cells were pre‐treated with Rg1 (40 μmol/L) with or without Ly294002 (10 μmol/L) for 2 h, and then exposed to G&P with 40 μmol/L Rg1 treatment with or without Ly294002 (10 μmol/L). A, Cell viability in different treatment groups (control; Glu + Pal; Rg1 + Glu +Pal; Ly294002 + Rg1 + Glu + Pal); n = 5 per group. B, LDH release in culture medium in each group; n = 5 per group. C, Cleaved caspase‐3 expression in each group tested by Western blotting; n = 5 per group. *P < .05 vs control group, ^#^ *P* < .05 vs Glu + Pal group, ^&^ *P* < .05 vs Rg1 + Glu +Pal group

We demonstrated that PI3K/AKT and Nrf2 pathways were activated by Rg1. We used PI3K inhibitor Ly294002 to treat H9C2 cells in the presence or absence of Rg1 and/or G&P to evaluate whether the PI3K/AKT pathway had a significant role in Rg1 defence against G&P damage. We found that Ly294002 partially reversed the protection of Rg1 against G&P injury as indicated by cell viability and LDH release (Figure 5A,B). Western blotting results revealed that H9C2 cells exposed to G&P had a significant increase in cleaved caspase‐3 expression compared with the control group, while those exposed to Rg1 had reduced cleaved caspase‐3 expression compared with the G&P group. This effect was partially reversed by Ly294002 (Figure 5C).

### 3.6 AKT/GSK‐3β/Nrf2 pathway plays an important role in Rg1 action against G&P‐induced H9C2 cell injury

**Figure 6:**
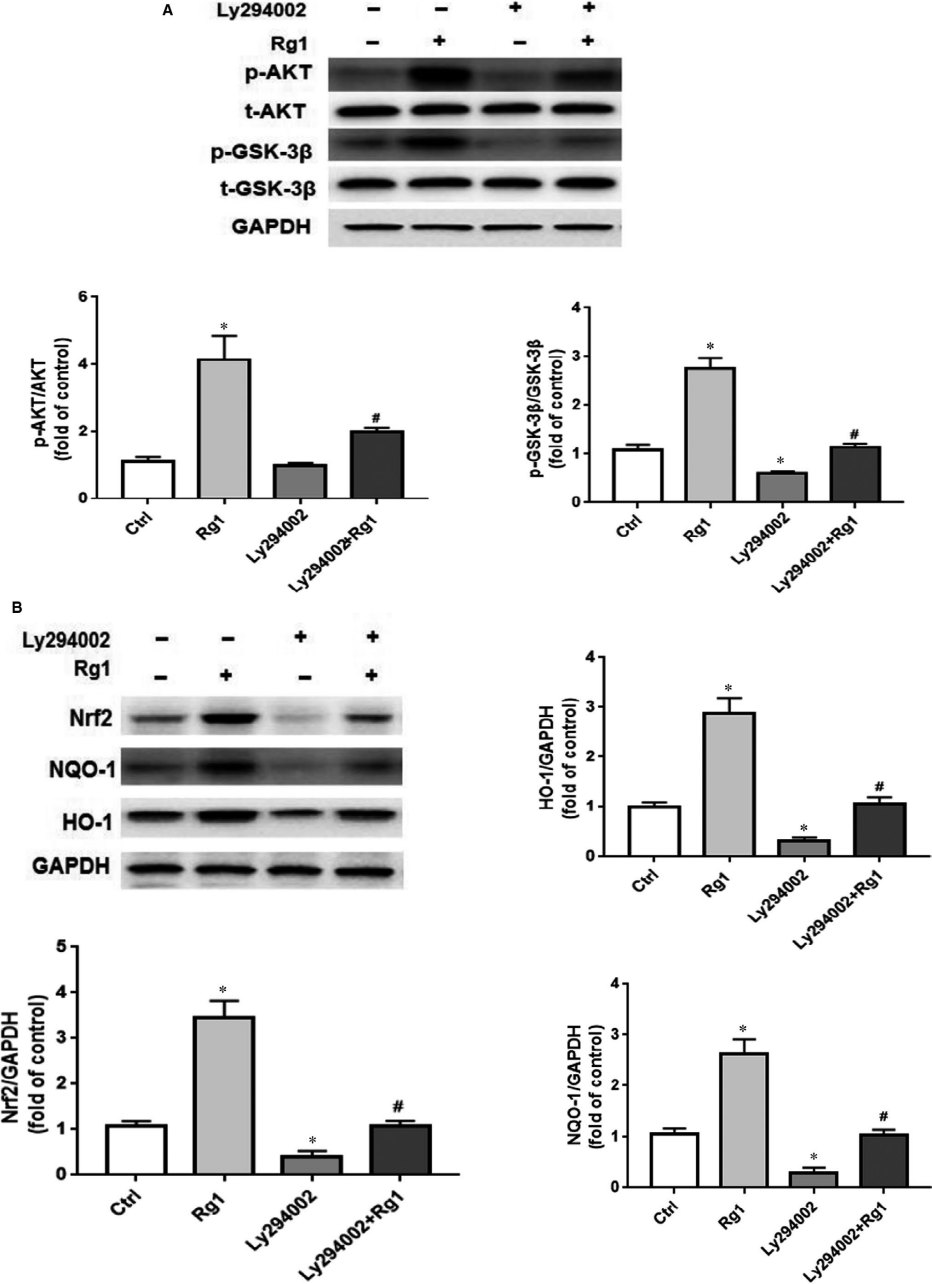
AKT/GSK‐3β/Nrf2 pathway plays an important role in Rg1 protection against G&P‐induced H9C2 injury. H9C2 cells were pre‐treated with Ly294002 (10 μmol/L) for 2 h, then co‐treated with Rg1 (40 μmol/L) for another 24 h. A, Expression of p‐AKT, t‐AKT, p‐GSK‐3β, t‐GSK‐3β in different groups (control; Rg1; Ly294002; Ly294002 + Rg1) tested by Western blotting; n = 5 per group. B, Nrf2, HO‐1, and NQO‐1 expression in each group (control; Rg1; Ly294002; Ly294002 + Rg1) tested by Western blotting; n = 5 per group. *P < .05 vs control group, #*P* < .05 vs Rg1 group

The transcription factor Nrf2 is an essential downstream target of the PI3K/AKT pathway.^15^, ^16^ As our previous results showed, AKT/GSK‐3β and Nrf2 pathways might be involved in the effect of Rg1 on G&P damage; we assessed the relationship between AKT/GSK‐3β and Nrf2 pathways. We identified a significant decrease in p‐AKT and p‐GSK‐3β expression after Ly294002 and Rg1 treatment compared with Rg1 group (Figure 6A). The same pattern was found in the expression of Nrf2, HO‐1 and NQO‐1 (Figure 6B).

## 4 DISCUSSION

**Figure 7:**
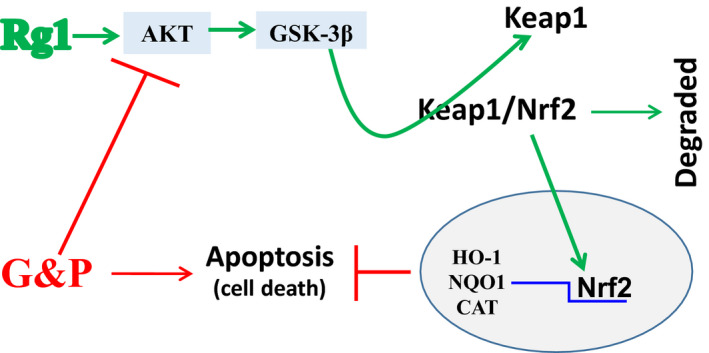
Schematic illustration of the mechanism by which Rg1 protects against G&P‐induced H9C2 injury. G&P could inhibit AKT/GSK‐3β pathway and induce H9C2 cell death, whereas Rg1 could activate AKT/GSK‐3β pathway by phosphorylation, which in turn dissociates Nrf2 from KEAP1 (Kelch‐like ECH‐associated protein 1), transposes into the nucleus, and recognizes the appropriate antioxidant response element (ARE) sequence. As a result, it initiates the transcription of a series of antioxidative genes harboring ARE in the promoter region, including HO‐1, NQO1 and CAT. These antioxidant products protect cells against oxidative stress‐induced apoptosis

Our previous study revealed that apoptosis plays an important role in the development of DCM in rats.^7^ In this study, we identified that Rg1 is non‐toxic to H9C2 cells at a dose of no more than 40 µmol/L (Figure 1A), and the protection conferred by Rg1 on H9C2 cells that are exposed to G&P is dose dependent (Figure 1B,C). We also identified that apoptosis plays an important role in G&P damage, and Rg1 alleviates G&P‐induced apoptosis (Figure 2A,B). In addition, Rg1 reduced G&P‐induced ROS formation and increased intracellular antioxidant enzyme activity (SOD, CAT and GSH‐Px activities) (Figure 3). We also found that Rg1 could activate AKT/GSK‐3β/Nrf2 pathway (Figure 4, Figure S1) and partially abolish G&P injury (Figure 5). PI3K inhibitor Ly294002 also down‐regulates Nrf2 activation (Figure 6) and partially reverses the protective effects of Rg1. Thus, Rg1 provides a protective effect against G&P damage in H9C2 cells that is partially AKT/GSK‐3β/Nrf2‐mediated. All the above‐assumed hypotheses are summarized in Figure 7, but remain to be examined in our future work.

Rg1 is one of the most important active components in ginseng extract, which has substantial medicinal value and physiological activity. The protective effects of Rg1 on the cardiovascular system have been confirmed by many studies.^7^, ^17^, ^18^ Several studies have shown that Rg1 has protective effects on diabetes‐induced cardiomyopathy.^7^, ^19^ Protective effect of Rg1 on the heart has been shown to be anti‐apoptotic and anti‐oxidative stress.^17^, ^20^ Our previous study reported that Rg1 ameliorates diabetic cardiomyopathy by inhibiting endoplasmic reticulum stress‐induced apoptosis in a streptozotocin‐induced diabetic rat model.^7^ To uncover underlying mechanism through which Rg1 protects against cardiac injury induced by diabetes, we mimicked diabetic conditions by culturing H9C2 cells in high glucose/palmitate and found that Rg1 showed significant protective effect on G&P damage by suppressing cell apoptosis (Figure 2) and ROS production (Figure 3A) and increasing intracellular antioxidant enzyme activity (Figure 3B‐D). This result agrees with what was observed in type 1 diabet**es** animal models.^20^

PI3K/AKT is an important insulin signal transduction pathway. PI3K/AKT signal pathway is closely related to apoptosis.^21^, ^22^ After PI3K is activated, it acts on the second messenger on the cell membrane, combining with AKT, and promoting AKT activation. The latter can regulate multiple transcription factors through phosphorylation, especially GSK‐3β, and is anti‐apoptotic.^23^, ^24^, ^25^ AKT activation is necessary for the phosphorylation of GSK‐3β, which serves as a potential modulator of cardiomyocyte apoptosis.^25^, ^26^ PI3K/AKT signalling pathway is closely related to DCM.^8^, ^27^ In the diabetic state, the PI3K/AKT signalling pathway is inhibited, resulting in metabolic dysfunction, myocardial inflammation, apoptosis, contraction dysfunction and finally DCM.^28^ Hyperglycemia can cause myocardial oxidative stress, activate a large number of cytokines, regulate myocardial cell function and apoptosis through AKT pathway,^29^ enhance PI3K activity to prevent cardiac remodelling, protect cardiac function in diabetic mice, and inhibit PI3K activity, which can accelerate the occurrence of DCM.^30^ In summary, PI3K/AKT signalling pathway is closely related to the occurrence and development of DCM, and regulating PI3K/AKT signalling pathway has become an important target to prevent DCM.^31^, ^32^ The protective effects of Rg1 on human hepatoma cell line HepG2,^33^ hypothalamic neurons^34^ and chondrocytes^35^ are related to the activation of PI3K/AKT signalling pathway. Thus, we have been used that Rg1 ameliorates G&P‐induced damage via the PI3K/AKT pathway in H9C2 cells. We found that Rg1 significantly increase**d** the phosphorylation of AKT and GSK‐3β (Figure 4A). Moreover, we used the PI3K inhibitor Ly294002 to confirm the role of the PI3K/AKT pathway in Rg1 protection against G&P injury. As expected, Ly294002 partially blocked Rg1 protection against G&P‐induced injury (Figure 6). These findings suggest that Rg1 promotes cell viability by activating the PI3K/AKT signalling pathway.

A number of studies have confirmed that the loss of Nrf2 aggravates diabetes‐induced cardiomyopathy.^36^, ^37^ Therefore, Nrf2 signalling pathway plays a crucial role in the inhibition of diabetes‐induced cardiac damage. Under normal physiological conditions, Nrf2 is sequestered by Kelch‐like ECH associating protein 1 (KEAP1) in the cytosol and degraded by proteasomes. Nrf2 is rapidly up‐regulated in response to hyperglycemia stress and then translocates to the nucleus to increase the transcription of oxidative stress response genes, such as *HO‐1*, *NQO‐1* and *CAT*.^38^ Moreover, several studies have demonstrated that Nrf2 may be up‐regulated by activation of AKT and GSK‐3β,^39^, ^40^, ^41^ and the nuclear translocation of Nrf2 requires the activation of the PI3K/AKT pathway.^42^, ^43^ In the present study, Rg1 treatment increased the phosphorylation of AKT and GSK‐3β, as well as the expression of Nrf2 (Figure 4), and ameliorated G&P damage by activating the AKT/GSK‐3β pathway (Figure 5). Moreover, Rg1 could significantly up‐regulate the expression of Nrf2 protein and its downstream genes *HO‐1* and *NQO‐1* to inhibit the oxidative stress injury induced by G&P (Figure 4B), whereas PI3K inhibitor Ly294002 decreased the levels of Nrf2 expression and its downstream gene expression (Figure 6B). These results indicated that Rg1 treatment exerts anti‐apoptotic effects by activating the AKT/GSK3β/Nrf2 signalling axis in H9C2 cells.

## 5 CONCLUSIONS

In conclusion, we demonstrated that Rg1 inhibits and improves G&P injury in H9C2 cells. Our results indicate that G&P induce significant H9C2 cell death, and this is substantially reduced by Rg1 treatment in vitro. The mechanisms involved in the therapeutic role of Rg1 in primary cardiomyocytes and an in vivo type 2 diabetes model are yet to be elucidated. Further studies are required to identify the role of Nrf2 in the protection of Rg1 against G&P‐induced damage, and define if the results of this study could be translated to in vivo diabetic models, especially using specific gene knockout mice.

## CONFLICT OF INTEREST

Haitao Yu, Juan Zhen, Yang, Jian Du, Jiyan Leng and Qian Tong declare that they have no conflicts of interest.

## AUTHOR CONTRIBUTIONS

**Haitao Yu:** Data curation (equal); Methodology (equal); Software (equal); Writing–original draft (equal). **Juan Zhen:** Data curation (equal); Formal analysis (equal); Methodology (equal); Software (equal); Writing–original draft (equal); Writing–review & editing (equal). **Yang Yang:** Data curation (equal); Formal analysis (equal); Software (equal). **Jian Du:** Data curation (equal); Formal analysis (equal); Investigation (equal). **Jiyan Leng:** Conceptualization (equal); Project administration (equal); Resources (equal); Supervision (equal); Validation (equal). **Qian Tong:** Project administration (equal); Resources (equal); Supervision (equal); Visualization (equal).

## Supporting information

Figure S1

## Data Availability

The authors declare that the data in this article are available.
