# Supplementary figures and images for: Rg1 protects H9C2 cells from high glucose‐/palmitate‐induced injury via activation of AKT/GSK‐3β/Nrf2 pathway
Source: J Cell Mol Med. 2020 Jun 16;24(14):8194–205. doi: 10.1111/jcmm.15486 (PMC7348154; doi:10.1111/jcmm.15486)

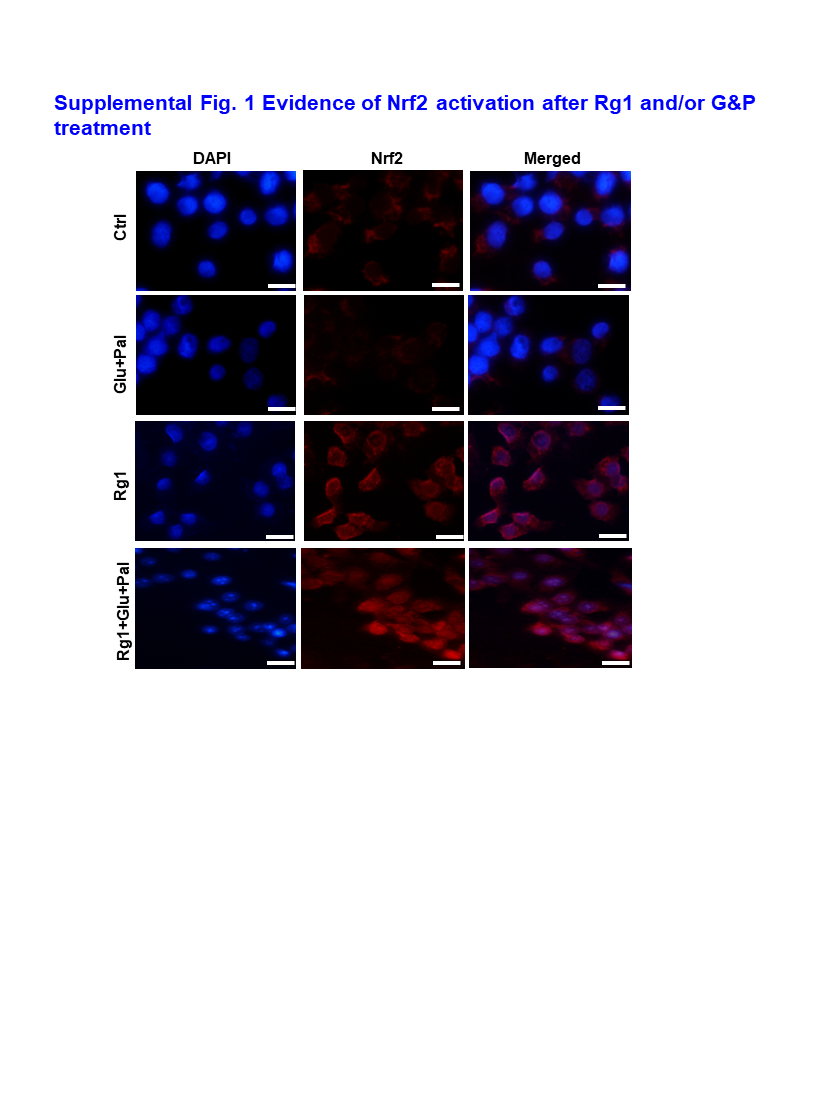

Supplement: Supplementary file 1 — Figure S1 [file JCMM-24-8194-s001.TIF]
